# A Comparative Analysis of the Early and Late Complication Rates and the Effect of Calcification on the Efficacy of MANTA and ProGlide Vascular Closure Devices

**DOI:** 10.7759/cureus.48092

**Published:** 2023-11-01

**Authors:** Abdul Hakeem, Mojahid Najem, Zakir Khokher, Arindam Chaudhuri

**Affiliations:** 1 Vascular Surgery, Bedfordshire-Milton Keynes Vascular Centre, Bedfordshire Hospitals NHS Foundation Trust, Bedford, GBR

**Keywords:** pseudoaneurysm, femoral artery calcification, vascular access procedures, manta vascular closure device, femoral artery access, large bore arterial access, perclose proglide suture mediated closure system

## Abstract

Introduction

The Perclose ProGlide and, more recently, MANTA Large-Bore Closure Device are commonly used vascular closure devices (VCDs) for managing large-bore vascular access haemostasis. The extent of calcification in the common femoral artery (CFA) plays a crucial role in choosing between these devices. ProGlide may face challenges with anterior calcification, while MANTA may have issues with posterior calcification. Our study compared their effectiveness, adjunct usage, calcification impact and early/late complications.

Methods

A retrospective analysis of procedures involving large-bore CFA access from 2017 to 2022 was conducted. Closure was grouped according to VCD as Group A (ProGlide) and Group B (MANTA). Calcification was designated as anterior and posterior and combined on pre-operative computed tomography angiography along 10 mm segments with 0.625 mm slice thickness. The success of haemostasis was graded as Grade 1 (haemostasis without adjuncts), Grade 2 (haemostasis with adjuncts) and Grade 3 (failed haemostasis needing rescue); Grades 1 and 2 were pooled as successful haemostasis. Statistical analysis was undertaken in Minitab 21 for Windows, particularly analysing calcification and its impact on the success of haemostasis.

Results

We evaluated 370 large-bore CFA accesses, distributed across two groups: Group A(64.9%, n=243) and Group B (35.1%, n=127), for a total of 205 endovascular procedures (93.1% (191) EVAR and 5.3% (11) TEVAR). The mean age was 74.9±8 years, predominantly males (88.2%, n=181). The average body mass index (BMI) was 28±5.8, with 20.9% (43) individuals having diabetes and 18.5% (37) current smokers. The mean sheath size OD was 16±2.5, with 4.5% (11) re-do groins in Group A and 6.2% (8) in Group B.

Successful haemostasis was achieved in 91.8% (n=223) in Group A (44.8%, n=109 Grade 2) and 90.5% (n=115) in Group B (21%, n=27 Grade 2). Rescue operations were needed in 8.2% (20) in Group A and 9.1% (12) in Group B. Pseudoaneurysms developed more commonly in Grade 2 haemostasis with 9.9% (11) in Group A and 1.6% (2) in Group B (p=0.3).

Anterior calcification was observed in 14.8% (36) in Group A and 18.8% (24) in Group B. In comparison, posterior calcification was present in 62.5% (152) in Group A and 66.9% (85) in Group B. Notably, calcification did not significantly impact haemostasis (p=0.79). Additional VCD deployment was necessary due to device failure in 4.5% (11) cases in Group A and 1.5% (2) cases in Group B.

Conclusion

The overall success rate was comparable between the two groups. However, Group A required more adjuncts to achieve successful haemostasis. The site of calcification did not impact the efficacy of closure devices. Pseudoaneurysm formation was more frequent when adjuncts were needed.

## Introduction

Haemostasis following large-bore femoral access for endovascular procedures, typically involving sheath sizes exceeding 12Fr, necessitates meticulous closure techniques to mitigate complications [1]. Closure methods often incorporate vascular closure devices (VCDs) [1], which encompass two main types: suture-mediated closure devices (SMCDs), like Perclose ProGlide® (Abbott Vascular, Abbott Park, IL, USA), and plug-based VCDs, such as MANTA® (Teleflex, Wayne, PA, USA), hereafter referred to as ProGlide and Manta, respectively.

ProGlide typically requires at least two devices for sheath diameters exceeding 8Fr [2,3]. These are deployed in an X pattern at the procedure's outset, with the suture left in situ and closed at the procedure's conclusion [4]. If bleeding occurs, additional ProGlide devices or plug-based VCDs can be deployed [4,5]. While calcification is considered a predictive factor for failure of haemostasis [5], the precise impact of calcification site on ProGlide closure remains inadequately explored. It is believed that anterior calcification may compromise suture integrity during closure, possibly necessitating supplementary manoeuvres.

By contrast, the plug-based VCD MANTA comprises an absorbable footplate and a collagen plug deployed at the procedure's conclusion to internally cover and seal the arteriotomy with collagen [6]. MANTA is explicitly designed for sheath sizes ranging from 12Fr to 25Fr [7]. In cases of failed haemostasis with MANTA, rescue options are limited. Research has suggested that anterior calcification might lead to improper footplate approximation and subsequent bleeding, while some studies propose a role for posterior calcification in haemostasis failure [8,9].

Complication rates reported in the literature vary widely, with ProGlide-related complications ranging from 4% to 14.7% and MANTA-related complications ranging from 10% to 20% [1,9-11]. However, a common issue in the literature is the classification of complications according to the Valve Academic Research Consortium-2 (VARC-2) criteria, which do not adequately consider the technical aspects of the studied devices. One study reports a specific complication rate of 7.6% for ProGlide, with higher incidence for sheath sizes exceeding 21Fr compared to those below 21Fr (17.2% vs. 4.9%) [5].

Similarly, calcification is typically categorised as mild, moderate or severe, with severe calcification defined as occupying >180 degrees of the arterial circumference [5,10]. One study defines anterior and posterior calcification by assessing calcification from the inguinal ligament to bifurcation using a horizontal line crossing the 3 to 9 o'clock position [12]. This classification is less commonly used and lacks comparison/applicability with MANTA. By contrast, the Manta Femoral Artery Calcification Score (MFAC) has been introduced, classifying calcification according to site and associating a score greater than three with inadequate haemostasis in MANTA cases [9].

Existing studies lack consistency in defining device and groin-related complications, making standardised haemostasis grading systems challenging. Therefore, there is a pressing need for a comprehensive classification system that incorporates technical aspects and haemostasis types in VCD research. This study adopts the classification system introduced in the MANTA group [13] to evaluate access-related complications for both ProGlide and MANTA devices. Our goal is to identify complications linked to VCDs and examine the influence of calcification site and extent. By analysing calcification's impact, we aim to enhance our understanding of its role in closure device effectiveness and offer guidance for clinical decision-making in large-bore vascular access procedures.

## Materials and methods

This retrospective analysis of a prospective database aimed to evaluate patients who underwent vascular procedures, specifically endovascular aortic aneurysm repair (EVAR), thoracic endovascular aneurysm repair (TEVAR) and popliteal endovascular aneurysm repair (PEVAR) from January 2017 to June 2022. Data were extracted from the National Vascular Registry (NVR) database. Those who underwent endovascular repair with open femoral access or lacked radiologic imaging were excluded. All patients had bilateral femoral access, and groins accessed with sheath sizes <12 Fr were also excluded from the analysis. All percutaneous accesses were ultrasound-guided and performed by experienced surgeons.

Data collection

**Figure 1:**
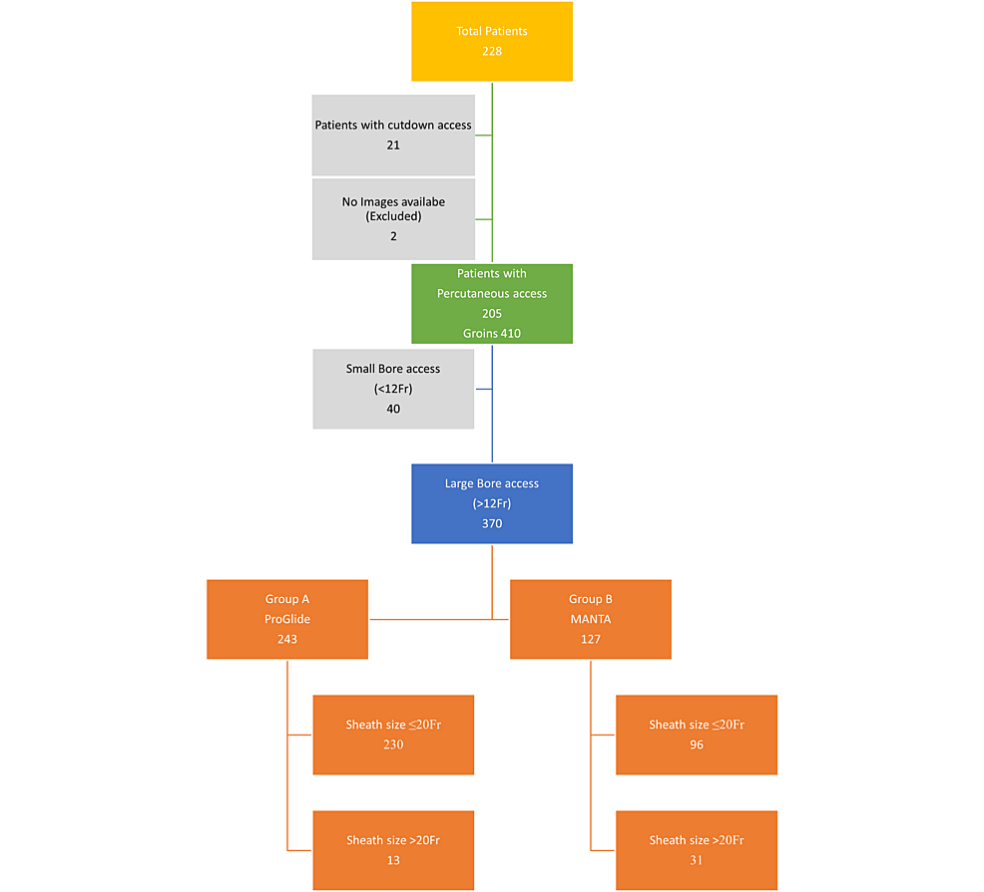
Selection flowchart, type of groin and sheath size distribution The data are represented as N (number).

Demographic and primary patient data were collected from the NVR, while technical aspects of the procedures, including the type of VCD used and any adjunctive procedures performed (e.g. cutdown and embolectomy), were obtained from operative records. Data were collected for all large bore accesses (>12 Fr) after meeting the exclusion criteria. The sheath size was further grouped as ≤20Fr and >20Fr as described in the literature [11] (Figure 1).

Classification of calcification

**Figure 2:**
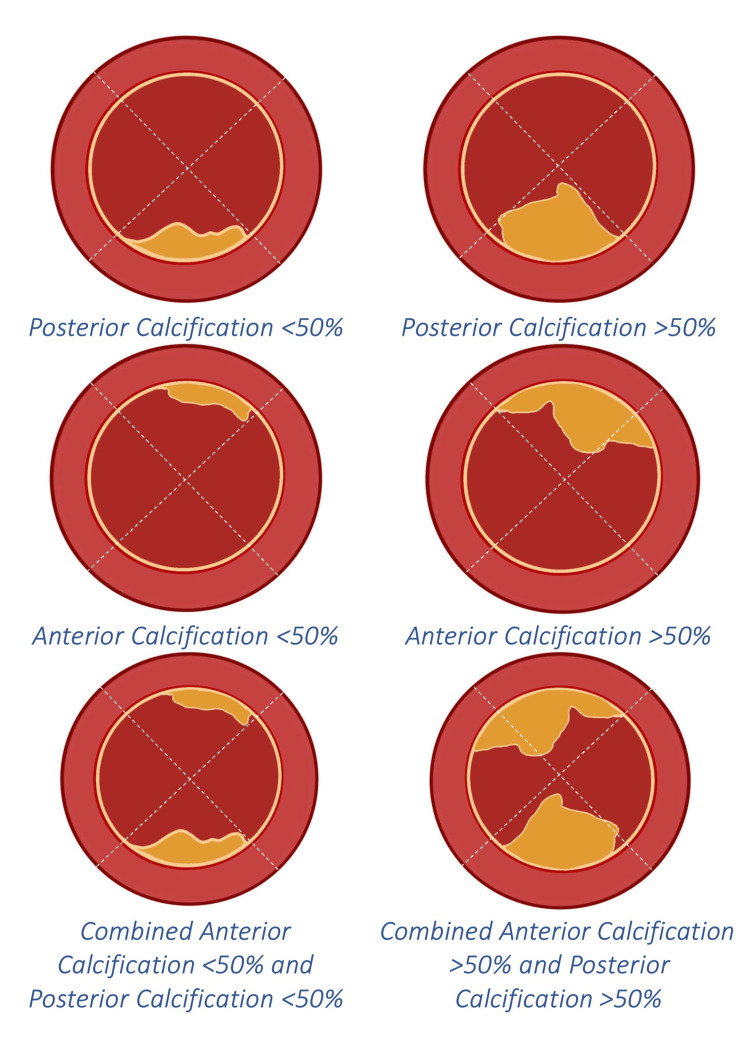
Classification of calcification Calcification was measured with a CTA at 10 mm intervals with a slice thickness of 0.625 mm, and an X pattern was delineated in the axial section, specifically targeting the region proximal to the bifurcation of the CFA. The picture is the author's creation. CTA: computed tomography angiography, CFA: common femoral artery

The common femoral artery (CFA) depth was assessed using pre-operative computed tomography angiography (CTA) scans by a single operator trained to evaluate the parameters accurately and avoid inter-operator bias. Calcification extent was measured along 10 mm segments with a slice thickness of 0.625 mm, proximal to the CFA bifurcation. An X pattern was marked within the CFA just above its bifurcation, and calcification was classified by site and extent on a quadrantic assessment accordingly. The grading of calcification included the following categories: no calcification, <50% calcification and >50% calcification (Figure 2). When calcification was in both the anterior and posterior quadrants, it was labelled as combined calcification; this was analysed separately to measure its significance.

Classification of access sites and haemostasis success

The access sites were categorised into groups, Group A and Group B, based on the type of VCD employed. The primary study objective was to assess the attainment of successful haemostasis, classified into three grades as presented in Table 1, and investigate the influence of calcification on haemostasis.

**Table 1:** Grades of hemostasis [13] Safeguard® (Datascope Corp., Fairfield, NJ, USA). 2 BD™ PTFE Felt Pledgets (BD, Tempe, AZ, USA) VCD: vascular closure device; PTFE: polytetrafluoroethylene

Grade of haemostasis	Description
Grade 1	Successful haemostasis achieved without the need for any adjuncts
Grade 2	Successful haemostasis requiring adjuncts, such as an additional VCD, pressure dressing, Safeguard®^1^ or PTFE pledgets^2^
Grade 3	Haemostasis achieved with rescue operations like surgical cutdown and repair

The secondary study objective centred on examining late access site-related complications, specifically stenosis, pseudoaneurysm or thrombus formation. These complications were evaluated by postoperative CTA scans conducted within four to six weeks.

Groin definitions

The primary groin was termed for groin access with no previous attempt of percutaneous or cutdown. A re-do groin was termed when the groin was previously accessed by either the percutaneous or cutdown method.

Data analysis

The collected data were analysed using Minitab software (developed by the Pennsylvania State University, version 21 for Windows). Continuous data were expressed as mean and standard deviation, while categorical data were presented as counts and percentages. The correlation with calcification was evaluated using a classification and regression tree (CART) analysis technique. In addition, the CART approach was utilised to determine the correlation of variables, such as BMI, skin depth and sheath size, to haemostasis. Fisher's exact and chi-square tests were utilised for 2x2 tables containing categorical data,with statistical significance defined as a p-value less than 0.05.

## Results

This study included 205 out of 228 patients meeting the exclusion criteria. The patients were divided into Group A, with 243 groins, and Group B, with 127 groins. The average age was 74.9±8.1 years, with 88.2% (n=181) males. The mean BMI was 28±6 kg/m², with 18% (n=37) being current smokers/recent quitters, 68.8% (n=141) ex-smokers and 21% (n=43) having diabetes. The most common pathology was abdominal aortic aneurysm (AAA), accounting for 89.7% (n=184) of the cases, including ruptured AAA, while other pathologies included penetrating arterial ulcers (2.9%, 6) and thoracic artery aneurysms (2%, n=4). The primary procedure performed was EVAR in 93.1% (n=191) of the cases (Table 2).

**Table 2:** Baseline characteristics of the patients The data are represented as N (number), % (percentage) and mean±SD. A P-value of p<0.05 is considered significant. BMI: body mass index, AAA: abdominal aortic aneurysm, CIAA: common iliac artery aneurysm, EVAR: endovascular aortic aneurysm repair, FEVAR: fenestrated endovascular aortic aneurysm repair, PEVAR: popliteal endovascular aneurysm repair, TEVAR: thoracic endovascular aneurysm repair, DOAC: direct-acting oral anticoagulants

Category	Class	Group A	Group B	Total	p value
Age		75 ± 7.8	74.8 ± 8.5	74.9 ± 8.1	0.87
Gender	Male	117 (90%)	64 (85%)	181 (88.2 %)	0.31
	Female	13 (10%)	11 (15%)	24 (11.7%)	
BMI		28.02 ± 6.26	28.14 ± 5.2	28 ± 5.8 kg/m^2^	0.89
Smoker	Current or stopped within two months	23 (17.6%)	15 (20%)	37 (18.5%)	0.15
	Ex-smoker	94 (72.4%)	46 (61.3%)	141(68.7%)	
	Never smoked	13 (10%)	14 (18.6%)	27 (13.1%)	
Diabetes	Yes	23 (17.6%)	20 (26.6%)	43 (20.9%)	0.12
	No	107 (82.4%)	55 (73.3%)	162 (79%)	
Pathology	AAA (unruptured)	121 (93%)	56 (74.6%)	177 (86.3%)	-
	Ruptured AAA	4 (3%)	3 (4%)	7 (3.4%)	
	CIAA	0	2 (2.6%)	2 (0.9%)	
	Dissection	3 (2.3%)	2 (2.6%)	5 (2.4%)	
	Endoleak	0	1 (1.3 %)	1 (0.4%)	
	Penetrating arterial ulcer	1 (0.7 %)	5 (6.6%)	6 (2.9%)	
	Popliteal artery aneurysm	0	3 (4%)	3 (1.4%)	
	Thoracic artery aneurysm	1 (0.7 %)	3 (4%)	4 (1.9%)	
Procedures	EVAR	126 (97%)	65 (86.6%)	191 (93.1%)	-
	FEVAR	0	1 (1.3%)	1 (0.4%)	
	PEVAR	0	2 (2.6%)	2 (0.9%)	
	TEVAR	4 (3%)	7 (9.3%)	11 (5.3%)	
Anticoagulation and antiplatelets	DOAC	20 (15.3%)	12 (16%)	22 (10.7%)	0.006
	Dual antiplatelet therapy	4 (3%)	6 (8%)	10 (4.8%)	
	Mono-antiplatelet therapy	67 (51.5%)	44 (58.6%)	111 (54.1%)	
	Warfarin	9 (6.9%)	5 (6.6%)	14 (6.8%)	
	Nil	3 (2.3%)	6 (8%)	9 (4.3%)	
	Missing	27 (20.7%)	2 (2.6%)	29 (14.1%)	

There were no significant differences in the BMI and CFA depth in both groups. Most cases involved primary groins (Group A: 95.4%, n=232; Group B: 93.8%, n=119), with a small proportion of re-do groins (Group A: 4.5%, n=11: Group B: 6.2%, n=8). Group A had a mean sheath size of 15.4±2.2 Fr, and Group B had 17±2.6 Fr. A higher proportion of sheath sizes ≤20 Fr was observed in Group A (94.6%, n=230) compared to Group B (75.5%, n=96) (Table 3).

**Table 3:** Comparison of two vascular closure devices Group A (ProGlide) and Group B (MANTA) data are presented as N (number), % (percentage) and Mean±SD. A P-value of <0.05 is considered significant. CFA: common femoral artery, VCD: vascular closure device,​SD: standard deviation, PTFE: polytetrafluoroethylene

		Group A	Group B	
CFA depth		34.0 ± 13.2 mm	34.1 ± 14.4 mm	
Groins	Primary groins	232 (95.4%)	119 (93.8%)	
	Re-do groins	11 (4.5%)	8 (6.2%)	
	Total	243 (64.9%)	127 (35%)	p=0.507
Sheath size	Sheath size ≤20 Fr	230 (94.6%)	96 (75.5%)	p=0.000
	Sheath Size >20 Fr	13 (5.3%)	31 (24.5%)	
	Mean sheath size and SD	15.4 ± 2.2 Fr	17 ± 2.6 Fr	
Additional VCDs	No	232 (95.4%)	125 (98.5%)	p=0.131
	Yes	11 (4.5%)	2 (1.5%)	
Grades of haemostasis	Grade 1	114 (46.9%)	88 (69.3%)	p=0.000
	Grade 2	109 (44.8%	27 (21.2%)	
	Grade 3	20 (8.23%)	12 (9.5%)	
	Success vs. failure (p=0.79)			
Types of adjuncts	Additional ProGlide	6 (2.4%)	-	
	Angioseal	55 (22.6%)	-	
	Angioseal with Safeguard	17 (6.9%)	-	
	PTFE felt pledgets	4 (4.6%)	-	
	Pressure dressing	10 (4.1%)	11 (8.6%)	
	Safeguards	21 (8.6%)	16 (12.5%)	
	Covered stent	1 (0.4%)	-	
	None	129 (53%)	102 (80.3%)	
Chronic findings				
Pseudoaneurysm	Pseudoaneurysms in Grade 1 haemostasis	2 (1.6%)	3 (2.4%)	
	Pseudoaneurysms in Grade 2 haemostasis	11 (9.9%)	2 (1.6%)	
	Total	13 (5.6%)	5 (4%)	p=0.3
Other chronic findings	Seroma	1 (0.4%)	0	
	Occlusive thrombus	0	0	
	Non-occlusive thrombus	6 (2.6%)	2 (1.6%)	
	Chronic haematoma	8 (3.4%)	4 (3.2)	
	None	201 (87.7%)	114 (91.2%)	
	Missing	14 (5.7%)	-	

The assessment of the haemostasis success revealed that Grade 1 was achieved in 46.91% (114) in Group A and 69.3% (88) in Group B, with no significant difference when considering Grades 1 and 2 together (p=0.8). Grade 2 haemostasis was observed in 44.86% (109) in Group A and 21.2% (27) in Group B. Grade 3 haemostasis was similar in both groups (Table 3).

Various adjuncts were employed to achieve haemostasis, with a higher utilisation rate observed in Group A. Specifically, 32% (78) of patients in Group A required additional VCDs, while PTFE felt pledgets, Safeguards, pressure dressings and covered stents were used. Grade 2 haemostasis was achieved in Group B through pressure application in 21.2% (27) of cases (Table 3).

Pseudoaneurysms were observed in 5.6% (13) patients in Group A and 4% (5) in Group B (p=0.3) and more common in Grade 2 haemostasis (i.e., 9.9% (11) in Group A and 1.6% (2) in Group B). Seroma was present in 0.4% (1) of patients in Group A and none in Group B. Occlusive complication was not observed in either group (Table 3).

The distribution of calcification differed between the two groups; Group A had a lower rate of overall calcification (65%, 156), compared to Group B (68.5%, 87). However, the overall success of haemostasis was not significantly affected by calcification (p=0.79).

**Figure 3:**
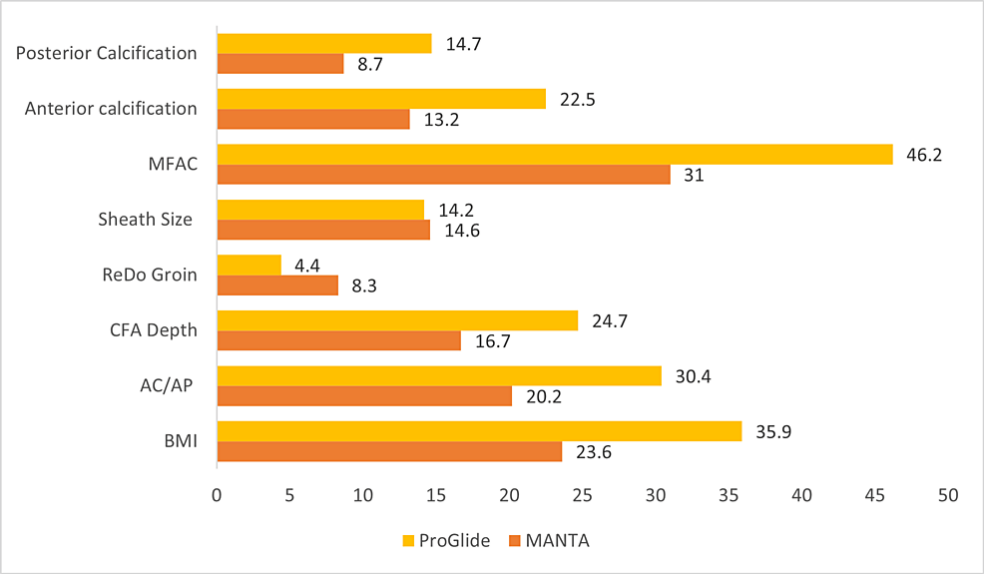
Graphic presentation of the correlation and regression tree (CART) analysis of factors influencing deployment success rates with ProGlide and MANTA. MFAC: MANTA femoral artery calcification

In Group A, 14.8% (n=36) had anterior calcification, while in Group B, 18.8% (n=24). By contrast, posterior calcification was more frequent in both groups, i.e. 62.5% (n=152) in Group A and 66.9% (n=85) in Group B (Table 4). Grade 3 haemostasis was seen more frequently in the presence of calcification in Group A compared to Group B; however, it was not statistically significant (p=0.79). The CART analysis indicated 5.8% contributory relevance of calcification in Group B (Figure 3).

**Table 4:** Distribution of calcification ^1^ Anterior calcification with/without other quadrants. ^2^ Posterior calcification with/without other quadrants. ^3^ Calcification in both anterior and posterior quadrants. ^4^ Calcification in only one quadrant. Data are presented as N (number) and % (percentage). Significance is considered if p < 0.05.

Site of calcification	Percent in quadrant		Group A	Group B
Anterior calcification^1^	Calcified >50%		6 (2.47%)	1 (0.79%)
	Calcified <50%		30 (12.35%)	23 (18.1%)
	No anterior calcification		207 (85.19%)	103 (81.1%)
Posterior calcification^2^	Calcified >50%		69 (28.40%)	11 (8.66%)
	Calcified <50%		83 (34.16%)	74 (58.27%)
	No posterior calcification		91 (37.45%)	42 (33.07%)
Combined anterior and posterior^3^	Any percent in any quadrant		31 (12.76%)	22 (17.32%)
Combined anterior and posterior^3^	>50% of each quadrant		4 (3.1%)	0
No calcification			87 (35.80%)	40 (31.50%)
Success on haemostasis based on the site of calcification				
		Haemostasis		
Isolated anterior^4^	>50%	Success	5 (83.3%)	1 (100%)
		Failure	1 (16.6%)	0
		Total	6	1
Isolated posterior^4^	>50%	Success	61 (88.4%)	9 (81.8%)
		Failure	8 (8.9%)	2 (18.1%)
		Total	69	11
Combined anterior and posterior^3^	>50% in both quadrants	Success	3 (75%)	0
		Failure	1 (25%)	0
		Total	4	0
No calcification OR <50% in any quadrant		Success	160 (93%)	105 (91.3%)
		Failure	12 (7%)	10 (8.6%)
		Total	172	115
The overall significance of calcification for haemostasis is p=0.79.				

The CART analysis identified critical predictors of successful haemostasis: a BMI >25.9 kg/m², anticoagulant and antiplatelet drugs and a CFA depth >2.5 cm. Furthermore, it revealed that the MFAC score >3 has more significance with the ProGlide device (46.2%) than with MANTA (31%). The BMI also substantially predicted ProGlide outcomes (35%) versus MANTA (24%). The impact of calcification was more significant in the ProGlide group (anterior 22.5% and posterior 14.7%) than in MANTA (13.2% and 8.7%) (Figure 3). 

We further analysed procedures for managing pseudoaneurysms. Six cases (33.3%) underwent open repair, two (11.1%) were treated with percutaneous repair using a stent-graft and two (11.1%) received thrombin injections. Eight (44.4%) did not require any specific intervention for their pseudoaneurysms, and settled spontaneously whilst managed on an observational basis. These findings highlight the diverse approaches to addressing pseudoaneurysms, with many cases managed conservatively without any procedural intervention (Table 5).

**Table 5:** Procedures performed for pseudoaneurysms Data are presented as N (number) and % (percentage).

Procedure	Frequency
Open repair	6 (33.3%)
Percutaneous repair (stent)	2 (11.1%)
Thrombin	2 (11.1%)
None	8 (44.4%)

## Discussion

The success rates and complications associated with ProGlide and MANTA devices have been extensively documented. However, a persistent challenge in VCD efficacy studies is the classification of complications, often limited by the VARC-2 criteria [14], categorising complications as major or minor bleeding without addressing nuanced technical aspects. Calcification's impact on VCD failure has gained attention, but classification discrepancies exist, and the specific location of calcification and its relationship with closure outcomes remain underexplored.

ProGlide studies report varying success rates, ranging from a 96.2% success rate [2] to conflicting findings regarding sheath sizes, from no significant difference in complications between ≤21 Fr and >21 Fr [15] to some studies reporting higher failure rates with sheath sizes ≥16 Fr, possibly due to device positioning challenges and an increased incidence of pseudoaneurysms [5,16]. Complications, assessed through angiography and VARC-2 criteria, reveal minor complications (14%) and pseudoaneurysms (1%) [17]. Calcification exceeding 50% impacts ProGlide success, but studies often lack precise methodological descriptions or location-based classification. By contrast, Manunga et al. identified calcification as an independent predictor, categorising it as anterior or posterior [12].

The SAFE-MANTA trial achieved a high technical success rate of 97.7% but did not grade haemostasis related to VCD deployment [7]. The MAnta Registry for Vascular Large-borE CLosure (MARVEL) study reported five device failures using the VARC-2 classification system [9]. Conversely, a study on plug-based devices noted access site bleeding rates of up to 7%, although it did not provide specific details about the control methods used [18]. Moccetti et al. found no significant relationship between calcification and failure rates but lacked methodological specifics and location-based calcification classification [8]. In addition, the MARVEL study introduced the MFAC score [9], associating a score ≥3 with risk but not distinguishing between anterior and posterior calcification as separate predictors.

Comparative studies between ProGlide and MANTA have reported varying complication rates. In one study, large-bore arteriotomies resulted in a 10% complication rate for MANTA and 6% for ProGlide, although specific groin-related complications were not defined [10]. Another study, utilising the VARC-2 classification system, documented complication rates of 10.7% for MANTA and 16.6% for ProGlide [11]. In a study by Maarten et al., which compared complications associated with ProGlide and MANTA VCDs, no significant differences in calcification were observed, but ProGlide was favoured in cases with calcification [10].

In this study, we introduced a graded haemostasis classification, demonstrating that both ProGlide and MANTA achieve optimal haemostasis outcomes. ProGlide often necessitates more adjuncts, while MANTA exhibits a higher success rate. CART analysis identifies higher BMI, greater CFA depth and anticoagulation usage as significant risk factors for ProGlide, with pseudoaneurysms being more frequent in cases with grade 2 haemostasis (Figure 3). We also categorised calcification by site and extent, correlating it with graded VCD-related complications. The ProGlide and MANTA groups exhibit distinct calcification patterns, with ProGlide showing higher posterior calcification rates and increased complications, while MANTA demonstrates superior haemostasis regardless of the calcification site. The MFAC scoring system predicts higher failure rates but does not elucidate the importance of the calcification site, displaying a stronger correlation with ProGlide compared to MANTA. In addition, the impact of BMI is less pronounced with MANTA, in line with prior studies highlighting MANTA's effectiveness in obese patients [19].

Study limitations

The study's limitations include being a single-centre study, a small sample size potentially impacting generalisability and statistical power, and possible exclusion of patients with missing data.

## Conclusions

Our comparative analysis of ProGlide and MANTA devices in vascular procedures using large-bore femoral arterial access revealed that both achieved successful haemostasis effectively. While ProGlide exhibited slightly higher failure rates, this difference lacked statistical significance, likely due to the utilisation of ultrasound guidance during femoral artery access, aiding precise puncture site selection regardless of calcification. These results underscore the importance of advancing percutaneous closure techniques and leverages application of imaging modalities like ultrasound to optimise closure outcomes by optimising access sites in the first place.

Furthermore, our study emphasised that scoring systems, while predictive of failure rates, may not fully capture the practical implications of calcification sites on device success rates. To gain a more comprehensive understanding of this relationship, a meticulously controlled randomised trial involving cases of arterial calcification is essential, though this may not be achievable in real-world practice. Overall, our findings contribute to the comparative effectiveness of these devices, offering valuable insights for clinicians in selecting appropriate vascular closure devices for vascular procedures.
